# Correction: Ruggiero et al. Golgi Phosphoprotein 3 Regulates the Physical Association of Glycolipid Glycosyltransferases. *Int. J. Mol. Sci.* 2022, *23*, 10354

**DOI:** 10.3390/ijms241713212

**Published:** 2023-08-25

**Authors:** Fernando M. Ruggiero, Natalia Martínez-Koteski, Viviana A. Cavieres, Gonzalo A. Mardones, Gerardo D. Fidelio, Aldo A. Vilcaes, Jose L. Daniotti

**Affiliations:** 1 Centro de Investigaciones en Química Biológica de Córdoba (CIQUIBIC), Consejo Nacional de Investigaciones Científicas y Técnicas (CONICET), Universidad Nacional de Córdoba, Córdoba X5000HUA, Argentina; m.natalia.martinez@unc.edu.ar (N.M.-K.); gfidelio@unc.edu.ar (G.D.F.); 2 Departamento de Química Biológica Ranwel Caputto, Facultad de Ciencias Químicas, Universidad Nacional de Córdoba, Córdoba X5000HUA, Argentina; 3 Facultad de Medicina y Ciencia, Universidad San Sebastián, Valdivia 5110466, Chile; viviana.cavieres@uss.cl (V.A.C.); gonzalo.mardonesc@uss.cl (G.A.M.); 4 CIQUIBIC (UNC-CONICET), Departamento de Química Biológica, Facultad de Ciencias Químicas, Universidad Nacional de Córdoba, Córdoba X5000HUA, Argentina

Viviana A. Cavieres and Gonzalo A. Mardones were not included as authors in the original publication [1]. Due to the sudden passing of Dr. Jose L. Daniotti, Dr. Cavieres and Dr. Mardones were unintentionally omitted from the submitted first version of the article. The corrected Author Contributions statement appears here. The authors apologize for any inconvenience caused and state that the scientific conclusions are unaffected. This correction was approved by the Academic Editor. The original publication has also been updated.

Fernando M. Ruggiero: conceptualization, methodology, data curation, formal analysis, writing—original draft, writing—review and editing, supervision, project administration, and investigation. Natalia Martínez-Koteski: methodology, data curation, formal analysis, writing—review and editing, and investigation. Viviana A. Cavieres: methodology and validation. Gonzalo A. Mardones: conceptualization, funding acquisition, project administration, resources, and supervision. Gerardo D. Fidelio: resources, writing—review and editing, and project administration. Aldo A. Vilcaes: conceptualization, methodology, resources, data curation, formal analysis, writing—review and editing, supervision, project administration, and investigation. Jose L. Daniotti: conceptualization, resources, supervision, and funding acquisition.
