# Supplementary material for: Golgi Phosphoprotein 3 Regulates the Physical Association of Glycolipid Glycosyltransferases
Source: Int J Mol Sci. 2022 Sep 8;23(18):10354. doi: 10.3390/ijms231810354 (PMC9499508; doi:10.3390/ijms231810354)
Supplement: Supplementary file 1 [file ijms-23-10354-s001.zip › ijms-1862244-supplementary.pdf]

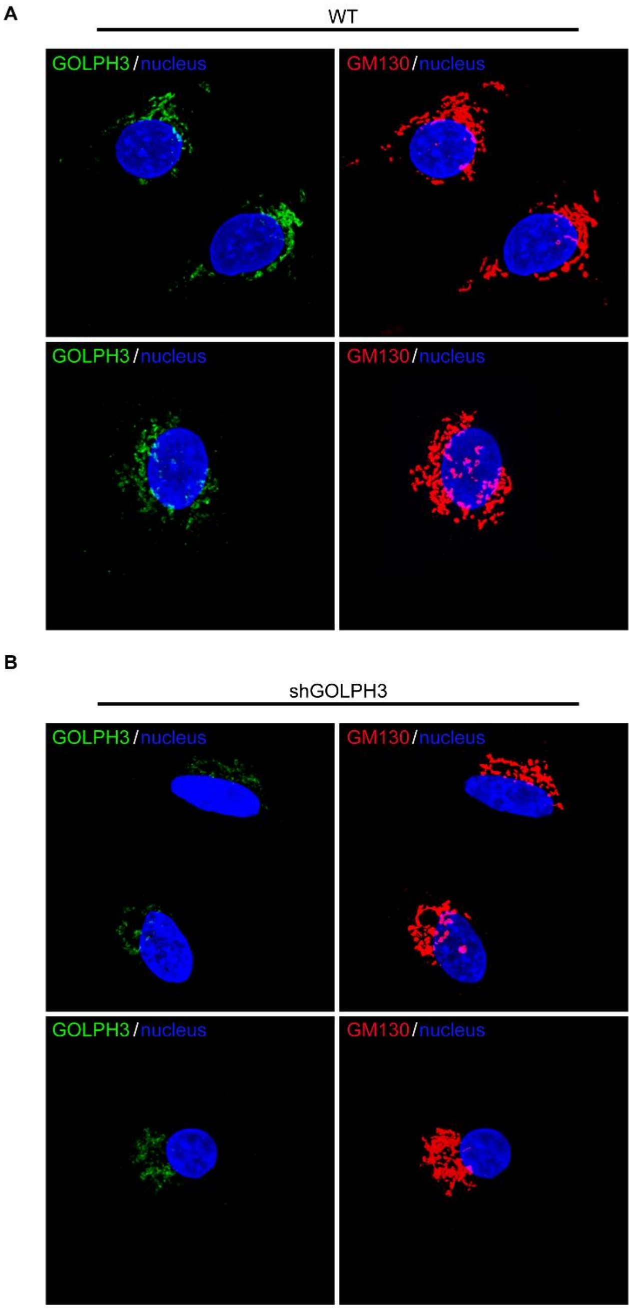

Knockdown of GOLPH3 modify the Golgi complex in MCF7 cell line.

Immunostaining showing the endogenous expression of GOLPH3 and GM130 in MCF7 cells transfected and non-transfected with specific shRNA to GOLPH3. All images result of the creates a 3D rendering from a stack. DAPI is in Blue.
